# Retraction: MABAC method for multiple attribute group decision making under single-valued neutrosophic sets and applications to performance evaluation of sustainable microfinance groups lending

**DOI:** 10.1371/journal.pone.0286614

**Published:** 2023-05-30

**Authors:** The PLOS ONE Editors

After this article was published, similarities were noted between this article and submissions by other research groups which call into question the validity and provenance of the reported results, and the adherence of this article to the PLOS Authorship policy. Further editorial assessment identified concerns regarding the integrity of the peer review process. In light of these issues, the *PLOS ONE* Editors retract this article [1].

HR did not agree with the retraction.
